# The role of transdisciplinarity for mineral economics and mineral resource management: coping with fallacies related to phosphorus in science and practice

**DOI:** 10.1007/s13563-022-00331-5

**Published:** 2022-08-23

**Authors:** Roland W. Scholz, Gerald Steiner

**Affiliations:** 1 Department for Knowledge and Communication Management, University of Continuing Education, Danube University Krems, Krems, Austria; 2 Department of Environmental Systems Sciences, Swiss Federal Institute of Technology (ETH), Zurich, Switzerland; 3 Institute for Advanced Sustainability Studies (IASS), Potsdam, Germany; 4 Complexity Science Hub Vienna (CSH), Vienna, Austria

**Keywords:** Transdisciplinarity, Self-referential transdisciplinarity, Mineral resource dynamics, Phosphorus, Scarcity fallacy, Simpson’s paradox, Cognitive didactics

## Abstract

Mineral economics is a genuine multidisciplinary field dealing with economic and policy matters related to the production, distribution, and consumption of mineral commodities. We discuss why the increasing complexity, ambiguity, ambivalence, and social contestation of subjects of mineral economics promote the participation of mineral economists in transdisciplinary processes. These processes relate (a) knowledge from targeted interdisciplinary processes and (b) mitigated discourses among different stakeholders to provide (c) a shared problem definition and to attain shared basic knowledge about problem transformation science and practice. We discuss known examples of misperceptions regarding minerals (phosphorus), such as an imminent scarcity threat, the incorrectly understood causations of the 2007/2008 price peak and present the *phosphorus ore-grades increased by 3.2% between 1983 and 2013 fallacies* (which is based on the Simpson’s paradox), and *only few countries have mineable reserves fallacy*. Here, we also illuminate motivations underlying several mineral economics–related misunderstandings. We argue that societally relevant questions require an honest mineral economics knowledge brokership. The example of the Global TraPs project, which targeted sustainable phosphorus management, is presented. Honest brokership to attain a clearinghouse function of science requires trust formation in society. We argue that this calls for increasing the understandability of relationships that are not well-understood, such as “if prices rise, so do stocks.” Wellmer and Becker-Platen’s feedback control cycle may be considered an example of how complex mineral economics can become and how challenging it is to be understandable to scientists from different disciplines and faculties as well as to practitioners whose knowledge may well be used to cope with the complexity of given problems. Thus, the present paper represents a plea for mutual learning between science and practice in order to understand the complex social and economic challenges of mineral resource dynamics.

## Introduction

Mineral economics is a genuine interdisciplinary field of a special type (Kesler et al. 2015; Radetzki and Wårell 2020; Rankin 2011; Rudawsky 2013; Tilton and Guzmán 2016). It connects the natural, social, and engineering sciences. The inner science knowledge integration (i.e., between disciplines) itself is a big challenge. For instance, John Tilton noted several requirements for a mineral economist:

One needs a good understanding of the constraints imposed … by existing technologies, economic considerations, governmental policies, and what is now widely recognized as the social license to operate. This requires some knowledge of geology and the earth sciences, finance, mining engineering, international relations and political science, and a host of other fields of relevance to mining and resources. In addition, … international trade, economic development, environmental economics, natural resource economics, and industrial organization … are often quite helpful as well in understanding the behavior of mineral industries and markets. (Tilton 2020, p. 274)

In this paper, we argue that, in transdisciplinary processes, such comprehensive interdisciplinary knowledge is a foundation beyond science by initiating processes of mutual learning between mineral economists and practitioners in a large range of fields from political affairs to community concerns (Scholz 2000; Scholz and Steiner 2015a). The behavior of mineral market actors, for example, depends on social values, norms, and personal priorities. We consider mining as an example. To understand how individual, cultural, and socioeconomic drivers might promote or hinder mining activities in certain ecologically and socially sensitive domains requires knowledge about the humanities as well. The role of values is essential in the opinion formation of mining projects. Usually, the proponents and opponents have different worldviews values and economic needs or pressures related to a mining project. In addition, public health concerns related to workers, communities, and the environment matter (Stephens and Ahern 2001). If societies become concerned about the inclusion of heavy metals in phosphorus (Marini et al. 2020; McLaughlin et al. 2021), new regulations may be applied to heavy-metal loads in P-fertilizers, which require additional chemical treatments that make mining in certain deposits uneconomic.

Mineral resource management has become a backbone of life and civilization that is embedded in a human-made, widely mineral resource–based material environment. This requires a broader perspective that goes beyond scientific disciplinary knowledge to incorporate experiential knowledge and the wisdom of practice. The integration of science and practice knowledge leads us to transdisciplinarity. This paper aims to demonstrate why practice and science are mutually dependent in sustainable mineral economics. This paper is of hybrid form. It discusses the need for transdisciplinary processes. This is done when considering a set of known fallacies such as the *scarcity fallacy* (Scholz and Wellmer 2013b, 2021) and the *phosphorus supply causes prices peak fallacy* (Weber et al. 2014) and two fallacies which have not been discussed that far much, i.e., the *only few countries have mineable phosphorus fallacy* and *phosphorus ore grades increased by 3.2% between 1983 and 2013*, which is due to Simpson’s paradox. The paper illustrates the value of transdisciplinarity by the example of Friedrich-W. Wellmer’s disciplinary, interdisciplinary, and transdisciplinary contributions.

### What is transdisciplinarity, and what does it mean?

Transdisciplinarity differs from interdisciplinarity (see Box 1). Transdisciplinarity provides added value by relating knowledge from science and practice to address and cope with ill-defined, complex, societally relevant challenges (Klein et al., 2001; Scholz et al. 2000; Scholz and Steiner 2015a). The term emerged in Europe (Jantsch 1970; Piaget 1972) and the USA (Mahan Jr., 1970) around 1970 because of insufficiencies in the integration and coherence of disciplinary and/or inner science knowledge (mode 1 knowledge) and for utilizing or generating scientific knowledge for understanding the increasingly complex challenges of the industrial society (Gibbons et al. 1994; Nowotny et al. 2001). From a mode 1 perspective, for instance, Einstein’s basic assumptions about the general theory of relativity or principles of quantum mechanics are incompatible with the basic postulate of particle physics (because, e.g., as quantum gravitation is missing in particular physics; see Ananthaswamy 2018; Hossenfelder 2017, 2018). This results in the call for a new, integrative, and consistent form of physics. These inner physical incompatibilities became a trigger and concern in regard to Nicolescu’s conception of transdisciplinarity. He conceived spirituality not only as an integrating level of all types of knowledge but also as a moral driver for aspiring to social justice as a goal of transdisciplinary processes in the frame of sustainability, for example, in the appreciation of indigenous knowledge (Nicolescu 2011, 2014).

**Fig. 1:**
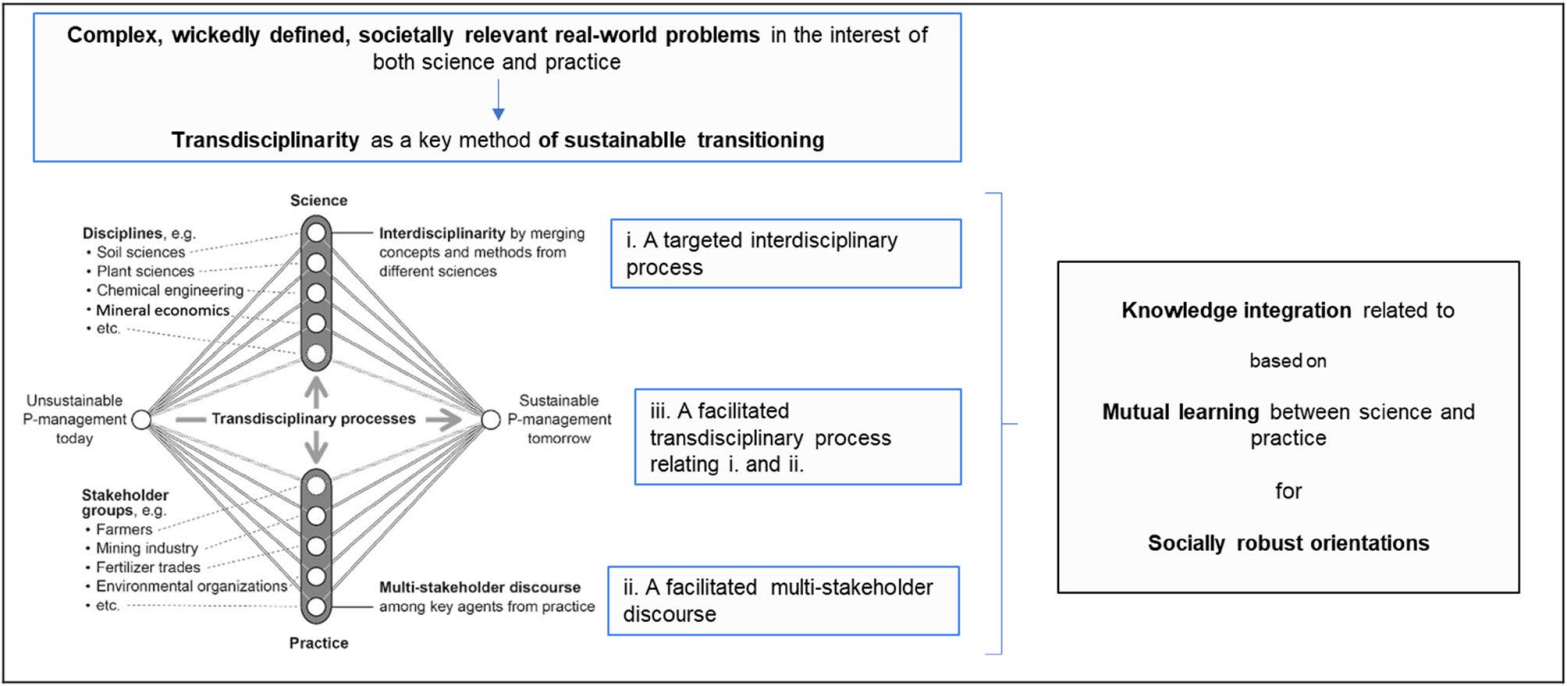
Transdisciplinarity as facilitation-based mutual learning process including a targeted interdisciplinary process and a facilitated stakeholder discourse for producing socially robust orientations (SoROs) for sustainable phosphorus (P) management (figure taken from Scholz and Le 2014)

We consider the *integration of concepts and methods* of different disciplines as interdisciplinarity. Game theory and hyphenated sciences (Sabelli 2006) such as biophysics or socio-psychology are interdisciplinary fields of scientific disciplines. However, the integration between sciences becomes difficult—if not impossible—when the rationales of reasoning and validation between disciplinary fields are incompatible. For example, the search for equilibria in economics cannot be merged with the evolution of technological innovations that may not be quantitatively represented, as Freeman stated earlier (1994). Therefore, the integration of certain fields of mineral economics often does not lead to interdisciplinarity but rather to multidisciplinarity, in which knowledge from different domains is constructed but fails to close with a full integration (Gordon and Tilton 2008). Transdisciplinarity, as we conceive it, aspires to mode 2, that is, the integration of epistemics from science and practice (Gibbons et al. 1994; mode 1 denotes inner science disciplinary and interdisciplinary). This particularly calls for reflecting on and mitigating the conflicts of goals, interests, values, and needs among stakeholders. Thus, mutual learning between science and practice offers consensus-building, mediation, capacity-building, and legitimization. In many cases, a facilitated multistakeholder discourse is beneficial (see Fig. 1, ii). We would add that transdisciplinarity may also take place in industrial settings. Large mining and IT companies have research departments that conduct and publish applied basic research as well as profit-driven departments at the frontline of the market whose knowledge may be synergized in strategic planning.

### Benefits of transdisciplinarity

The public often views extractive industries as scrupulous economic agents that endanger cultural heritage, social equity, and people’s work and health due to self-interests, as well as engaging in greenwashing, corruption, and other destructive activities (Barton 2022; Rae, Rouse, & Solomon, 2002). The degree of corporate responsibility differs between companies (MMSD 2002), and their behavior and corporate social responsibility (CSR) differ significantly between nations and according to situations and opportunities related to the “interaction of survival instinct at the notion of social license to operate” (Owen and Kemp 2013, p. 13). We argue that transdisciplinarity is the appropriate means for engaging community consent and utilizing CSR to develop a social license to operate when dealing (a) with the trade-offs between stakeholder interests, environmental and social responsibility, etc., and (b) the economic/market and political constraints of companies (Agudelo et al. 2019). This also challenges mineral economics, as a scientific domain, to provide proper models for integrating external costs and social welfare functions in a specific place or in general. Transdisciplinary projects on nuclear waste management (Krutli et al. 2010), sustainable phosphorus management (Scholz et al. 2014a, b), and mobility (Loukopoulos and Scholz 2004), among others, have demonstrated that socially robust orientations (SoROs; see Box 1) are effective main outcomes.

**Fig. 2:**
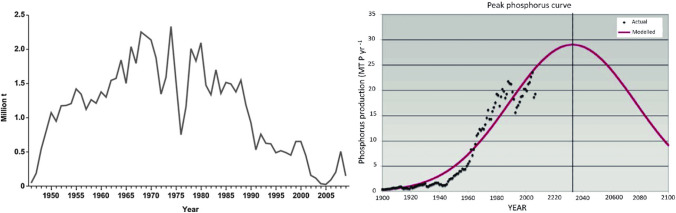
Production curve of guano phosphate on Nauru Island (Déry and Anderson 2007) and reserve-based peak phosphorus prediction by Cordell et al. (2009)

**Table:** 

Box 1 Friedrich-Wilhelm (Fred) Wellmer and transdisciplinarity — a boundary walker meets boundary objects Successfully coping with the complex, societally relevant, ill-defined, ambiguous, and frequently contested challenges of the twenty-first century calls for thorough, discipline-based interdisciplinarity and transdisciplinary processes (Scholz 2011). To achieve these, scientists must venture beyond their inner scientific comfort zones and become transdisciplinarians. During his professional life, Fred Wellmer took on different roles working at the interface of mineral science as a scientific field and as a participant in various societal interest and actor groups (Wellmer 2022). Doing so requires the ability to interact with stakeholders who have different roles and interests and who are concerned about, causing, or regulating challenges and contestation among key actors and other stakeholders in the context of often-sensitive issues related to the mineral supply chain. The costs and benefits of mining may be viewed as boundary objects. The value assigned to the external costs of mining in the frame of sustainability, as part of the total operating costs, is an issue that asks a boundary walker “to translate across culturally defined boundaries, for example, between communities of knowledge or practice” (Fox 2011, p. 70). Fred Wellmer has successfully done so by taking on these different roles. After earning a PhD in geology, Wellmer began his career as an exploration geologist with a mining background working for the Metallgesellschaft, the largest German company in this domain at that time. One of his assignments was the search for non-ferrous metal deposits in Canada, applying geological, geophysical, and geochemical methods. In addition to his practical exploratory work with colleagues from public research institutions, he initiated the development of geochemical tools to discover subtle halos and gradients to ore in situations where geophysical signals are masked (Schoell and Wellmer 1981; Wellmer et al. 1999). Other subjects of his work include the physical exploration of the Western Australian Callion gold deposit (Glasson et al. 1988) and the zinc–lead–copper deposits of the Toqui District in Chile (Wellmer et al. 1983). Later, however, Wellmer moved from exploration to geo-economic analysis. One analysis involved the (economic and, later, sustainable) evaluation of exploratory activities (Wellmer, Dahlheimer, & Wagner, 2008; Wellmer and Drobe 2018), particularly in regard to the optimal lifetime of a potential deposit (Wellmer and Scholz 2018). In this context, the conception and study of feedback control cycles (including the impacts of recycling and the substitution of minerals in product development through innovation) emerged. Another was geostatistics (Wellmer 2012) and geostatistic modeling (Wellmer and Giroux 1980) in connection with discovery as the economic output of exploration (Sames and Wellmer 1981). The uncertainties in the data as well as in the knowledge necessitate an in-depth understanding of economic and financial risk management. Wellmer exchanged his practice hat for a governmental (public agency) one when he worked at the Ministry of Economics and later as department head and president of the Federal Institute for Geosciences and Natural Resources (BGR, i.e., the German federal geological survey). Given his profound interest in the scientific foundations of mineral science, economics, and politics, he became a professor at the Technical University of Berlin in 1991 in the field of Raw Materials Policy and Mineral Economics, thus acknowledging his high-profile scientific contributions to the geosciences. One of Wellmer’s most seminal and groundbreaking contributions to ecological economics is the concept of the feedback control cycle (FeedbaCC; Wellmer and Becker-Platen 2002; see Fig. 2). The FeedbaCC is a boundary object par excellence. FeedbaCCs are always unique and specific as well as generic, but as boundary objects, they “are plastic enough to be adaptable across multiple viewpoints, yet maintain continuity of identity” (Star 1989). The societal, global perspective, and the global responsibility perspective certainly emerged from his international experience as an exploration geologist with a mining background and his insights as the leader of the BGR in the fundamental socioeconomic, technological transition from the industrial age to the digital society, and coping with multiple disruptive environmental, social, and cultural changes in the departure from the fossil age. The particular role of geosciences and geological services and the necessity of taking a system view that focuses on the resilience of human–environment interactions and the need to change the perspective from a life *on* Earth to a life *with* Earth have been saliently demonstrated by the 1999 book *Living with the* *Earth: Contributions of Geological Surveys for the Services to the Public and *. *Sustainable Development* (Wellmer and Becker-Platen 1999). Wellmer’s strong involvement in transdisciplinarity is part of this vision.	

The range of approaches to and definitions of science–practice collaboration is broad (Renn, 2021; Scholz 2011). We can observe an increasing differentiation between an approach in which scientists function as activists (Scholz 2020; Scholz and Wellmer 2021) promoting advocacy-based solutions and one where scientists with a real-world problem orientation serve all societal stakeholder groups that follow national institutions and human rights. In the following, we refer only to the second approach. This is because the proposed approach of transdisciplinarity much relates to agenda setting (i.e., joint problem definition), whereas later stages of monitoring, evaluating, and solution finding may allow for other forms of collaboration.

Mutual learning between science and practice forms the core of transdisciplinary processes. Thus, herein, we propose to illustrate how sustainability-oriented mineral economics can assist practice in more effectively coping with misunderstandings, misconceptions, and the challenges of practice and how mineral economics is challenged to contribute to sustainable-resource management in the real world.

We conclude this introduction by distinguishing between three ways that knowledge from science and practice can be integrated. The first is the functional mode of transdisciplinarity. Here, participating scientists and practitioners collaborate to better understand the complexity of a certain issue, e.g., increasing the energy efficiency of beneficiation as a step in mineral production. The knowledge that is generated helps practitioners to improve a mine’s production processes, whereas mineral scientists may better understand the interaction between certain stages and variables of the chemo-physical transformation processes in beneficiation (Steiner et al. 2015).

Second, from a democratic perspective, we aim to include all key stakeholders in a balanced manner. Stakeholders are actors who are responsible for, who are concerned, or who regulate important factors such as critical social and/or economic costs. Attaining a balanced stakeholder representation from among those who are not only willing but also capable of participating in a transdisciplinary process is a significant challenge and one that may, indeed, not be possible in certain political and/or cultural settings (Arenas et al. 2020; Mielke et al. 2017). However, we do not address this problem in detail here. In general, a democratic culture that embraces principles such as “accepting the otherness of the other” and evidence-based reasoning as part of the code of conduct is necessary.

Finally, we define self-referential transdisciplinarity. We speak about self-referential transdisciplinarity, if someone has acquired elaborated, mature knowledge in a field of science and of practice, and can explicitly synergize these two modes of knowledge. The idea is that individuals working in various roles and having different functions acquire different types of knowledge, rationales, and experiences. Friedrich-Wilhelm Wellmer (see Box 2), to whom the present special issue of Mineral Economics is dedicated, may be considered an example. Wellmer, professor at the Technical University of Berlin, conducted high-profile research and published papers specializing in geochemistry, geo-economic and geostatistical modeling, and raw materials and sustainability. He served as the head of the German Geological Survey from 1996 to 2002 and before that worked as chief exploration engineer of a large mining company in Canada, Australia, and other countries. Thus, he has worn different hats, and we may speak about self-referential transdisciplinarity in relation to him.

**Table:** 

Box 2 Guiding question and goals of the Global TraPs project The aim of the Global TraPs project is to engage key stakeholders through transdisciplinary mutual learning in building a human–environment, system-based understanding of the complete phosphorus supply and demand chain, identifying pools, sinks, and the underlying dynamics of flows, in order to jointly identify alternatives for use, reuse, and recycling through case study research with strategic stakeholders (see the NSSI Td pages). Answering the guidin*g* question, “What new knowledge, technologies, and policy options are needed to ensure that future phosphorus use is sustainable, improves food security and environmental quality, and provides benefits for the poor?” will lead to improved resource understanding and awareness, leading to sustainable P management and stewardship. The Global TraPs project aspired • To be a leading global learning forum for sustainable P use, management, and stewardship in providing an open discourse space for all stakeholders along the P supply chain in a transdisciplinary (joint, eye-level, transparent), complementary, and non-politicized arena • To define the current state of knowledge on phosphorus and its use, and what new knowledge is necessary to ensure sustainability over the whole *P* value chain from diverse case studies being conducted by partners in 2013 and 2014 • To define new technologies that are required in order to better process, use, and reuse phosphorus • To define the most valuable areas for policy intervention to ensure sustainable P use in the future (Taken from the Global TraPs web page: http://www.globaltraps.ch; November 3, 2021)	

For self-referential transdisciplinarity, it is important for the person who practices it to know which types of knowledge belong to which backgrounds and can, thereby, be applied to which types of problems. Transdisciplinary processes are different from those generated by the triple helix model (Etzkowitz and Leydesdorff 1995), which describes the collaboration between industry, politics, and government. One reason is that the model has been shaped mostly by an industrial and a capitalization of knowledge perspective (Etzkowitz et al. 1998). Transdisciplinarity, by contrast and as conceived by Wellmer and the authors of this paper, is seen as a tool for sustainable development (Wellmer and Becker-Platen 2002; Wellmer and Hakelüken 2015) that focuses on a resilient social system. This includes the allocation of balanced benefits and burdens for all stakeholder groups and requires some degree of commitment to inter- and intragenerational justice (Brundtland et al. 1987; WSSD 2002), thereby demonstrating how normative aspects are included in any sustainable action (Scholz 2017).

The structure of the paper is as follows. Part 2 reviews how questions of mineral economics first became the subject of transdisciplinary processes. With a mineral and phosphorus scarcity discussion, part 3 illustrates the central roles and functions that mineral economics may play to empower civil society and decision-makers to understand and meaningfully cope with problems such as scarcity and regulatory schemes, technology innovation, and others. In the discussion in part 4, several demands that have emerged in the course of transdisciplinary processes regarding phosphorus in relation to mineral economics are formulated.

## How mineral economics met transdisciplinarity

### Four milestones on the way to transdisciplinarity

Presumably, a number of mineral economists have practiced transdisciplinarity without knowing the term or realizing it. Thus, a subsequent description might be somewhat biased. Yet, undoubtedly, Friedrich-W. Wellmer is a pioneer in the development of transdisciplinarity in mineral economics. With respect to mineral economics and management, we want to mention four historical events that occurred along the way toward linking mineral sciences and environmental/sustainability sciences with transdisciplinarity.

First, environmental scientists have been impressively concerned about the Club of Rome’s “mineral scarcity soon” mission. The scarcity concept has become a key to life cycle and social resilience assessment (Dedeurwaerdere 2014; Graedel et al. 2015). The first author of this paper recalls clearly when Friedrich-W. Wellmer provided a drastically different message to the 2001 Congress “Life into tomorrow’s world — life cycle engineering and industrial “economics” at the Technische Universität Braunschweig, Germany.” He introduced the feedback control cycle (see below “What do transdisciplinary processes need from mineral economics?”), which drew the attention of at least those participants from the green side who were educated economists or trained in systems dynamics.

Second, as an impact, his presentation at the 2007 workshop on scarce raw materials addressed the challenge of structuring the field of non-energy mineral resource scarcity. Two camps learned from each other; on one hand, environmental scientists began to acknowledge the principle of “prices increase the stocks of minerals” (e.g., reserves or resources) and, thus, were challenged to think about the dynamics of mineral reserves and resources. On the other hand, those in the engineering camp had to acknowledge that phosphorus is a bioessential and non-substitutable element of life.

The two camps met when considering low-use efficiency and the environmental impacts related to it (Chen and Graedel 2016). Unfortunately, phosphorus use has a highly dissipative structure including run-offs and erosion. According to the Paracelsus principle of “not too little–not too much,” large amounts of phosphorus, particularly in aquatic systems, make the nutrient P a pollutant with severe environmental impacts (Scholz et al. 2014a, b).

The rule that when prices increase, stocks increase as well has a socioeconomic limit. If costs for a specific mineral become too high, we face scarcity. As phosphorus is non-substitutable, we may argue that mineral-resource scientists must also become sensitive to very long-term phosphorus supply security. Although it is distant and, fundamentally, beyond the human mind’s capacity, this may call for thinking about time frames of the magnitude of 100,000 years as a future window for the human species.

Third, to promote mutual learning between the two camps, i.e., practitioners and scientists, and to overcome positional and argumentative lock-in, the GlobalTraPs project for sustainable phosphorus management was initiated (Scholz et al. 2014a, b). This project addressed all the links in the supply chain including exploration and mining as two nodes, and it was a transdisciplinary project in the strongest sense as it included a co-leadership of practitioners and scientists at all levels of this four-year process of mutual learning. The practice side included key members from the international Industry Fertilizer Association (IFA), the US Geological Survey (USGS), the German Geological Survey (BGR), beneficiation technology providers (e.g., Outotech), and the International Plant Nutrition Institute (IPNI)—the international agronomy think tank. A wide range of governmental and environmental representatives including those from the UN Environment Program and Greenpeace complemented the practice side, as presented in Fig. 1. Scientists from a broad range of disciplines had to acknowledge the different dimensions of sustainability.

Questions about the future scarcity of phosphorus, the reliability, as well as the uncertainty of geological exploration on different scales (Vaccari et al. 2014) and the losses, the efficiency, and the external costs of mining (Watson et al. 2014) were core issues. The notion of losses was highly disputed, and the discourse revealed that the answer to the question of how much phosphorus is disappearing from the supply chain in the course of mining operations could not be answered. The costs of mining “leftovers” (above the cutoff grade) with difficult physical and economically risky access or the costs of depositing close below cutoff grade overshoot are difficult to access at local and global dimensions (Wellmer and Scholz 2018).

The fourth and final milestone, the April 2012 conference on “Life and Innovation Cycles in the Field of Raw Materials Supply and Demand: A Transdisciplinary Approach,” was organized by Friedrich-W. Wellmer at the end of his tenure as Le Studium Research Chair Guest Professorship at the Loire Institute for Advanced Studies and the French Geological Survey (BRGM) in Orléans, France (Le Studium, 2012). The conference dealt with the adaptation of tightened mineral supply and demand, discussing among other topics who is learning what from whom in the course of adapting mineral production to the social and geopolitical constraints of the twenty-first century.

## The role of mineral economics in transdisciplinary processes: overcoming fallacies and biases

According to a common understanding, science should serve as a societal clearinghouse of knowledge (Scholz 2011). This holds true for evaluating both the positive and negative aspects of an activity such as mining mineral commodities. Unfortunately, some questions about mineral economics, such as the environmental costs of mining or of utilizing a particular substance like mercury for specific purposes (Hylander and Goodsite 2006), the scarcity of minerals (Meadows et al. 1972; Tilton 2003), and mining’s social benefits are societally contested. This is due not only to multiple stakeholder disputes that might refer to different values, assumptions, and scenarios (of exposure) but also to the interdisciplinary nature, the complexity of the situations, and the multiplicity of system boundaries, e.g., of time, space, or actors, that do not allow for unambiguous modeling. Yet, as we will show below, fundamental errors that ignore basic mineral economics knowledge are sometimes committed (Scholz & Wellmer, 2016, 2021) and call for correction. From a societal—but also from a scientific perspective—economics and, thereby, mineral economics represent an important approach that may not only help companies to improve their return on investment but also help society to better understand the costs and benefits of mining activities. Mineral economists are often challenged to adapt their models to respond to societal questions. The increasing demand for the incorporation of (different) societal costs and social welfare functions can serve as an example of why mineral economists should participate in transdisciplinary processes. We illustrate this for the case of phosphorus.

### Several special characteristics of phosphorus

From a social resilience perspective, phosphorus has been judged the most important mineral at the present time as it is non-substitutable for food production and, therefore, for life (Wellmer and Scholz 2017). Since fully half of current food production is based on mineral fertilizer and phosphorus is one of the three major macronutrients, this is accurate. Unfortunately, we do not know when, or even whether, we may find a Haber–Bosch-like process to extract phosphorus from areas of oceans that have phosphate-saturated water, i.e., “phosphorite factories” (Pufahl and Groat 2017; Scholz and Wellmer 2019; Wellmer and Scholz 2017), should terrestrial mining costs increase critically in the very long run (e.g., several thousands of years ahead).

In contrast to nitrogen, it is unclear whether we can economically produce a Haber–Bosch-like procedure (which allows for ammoniac, NH3, recycling and makes nitrogen a renewable, infinite entity) for phosphorus extraction by developing an alternative for producing marketable phosphate rock. Potassium production from rocks and soluble salts can be supplemented utilizing sea resources but not yet due to the economic costs involved (Jena 2021). Currently, this functions only on a demonstration-plant scale (Ghara et al. 2014). A physical scarcity of potassium is less critical as its concentration in Earth’s crust at 2.41% is much higher than that of phosphorus at 0.09% (Binder 1999) (daily consumption of potassium is about a factor of 2 higher than that of phosphorus). The shortest mineral cycle in a phosphate rock-to-phosphate rock return is about 20,000 years in high-phosphate-laden seawater such as the Gulf of Mexico and, thus, significant from a human species development perspective (Scholz and Wellmer 2019).

In food production, the use of phosphorus has a high dissipative structure. In aquatic systems, large phosphorus concentrations from runoff, erosion, leaching, etc. have been known to be critical pollutants for decades (Goyette et al. 2018; Rabalais et al. 2009). Because of smallholder farmers’ difficulties accessing phosphorus, particularly in sub-Saharan Africa (Chebet et al. 2019; Njoroge et al. 2015), the economic mechanisms of its distribution are an important aspect of social justice and even human rights since it is a prerequisite for food production (Wellmer and Scholz 2017, 2022).

The non-substitutability of phosphorus challenges weak sustainable positions arguing that human capital (i.e., the ingenuity of the mind) may arbitrarily be a substitute for natural capital (Wolfensberger et al. 2008). Fortunately, phosphorus’s short- and mid-term physical availability, compared to other minerals, is quite generously secured; identified reserves cover about 288 years of current annual phosphorus consumption (food production accounts for more than 85% of consumption). The known resources, if completely recovered, would last for more than 1000 years (Jasinski 2022; Scholz and Wellmer 2013a). If we were to restrict recovery to Morocco’s sub-Saharan surface mines (Scholz and Wellmer 2016; van Kauwenbergh 2010) and the US’s western phosphate fields (Moyle and Piper 2004), huge amounts of phosphate rock of close to resource quality would still be available. Moreover, phosphorus deposits can be found in more than a hundred countries (Orris and Chernoff 2002), and most of these have some economic potential (Orris and Chernoff, 2004). However, current economic options for large-scale mining and/or a lack of willingness to mine (also known as NIMBY for “not in my backyard”) restrict mining in many places. For instance, the USSR Ministry of Fertilizer Industry had plans to mine the large Estonian Virumaa phosphate reserves, but these were blocked in 1987 (during the glasnost period of Soviet policy) by Estonian scientists and students with “a resolution that accused the Ministry of Fertilizers of neglecting the ecological and social effects of mining” and surveys that showed three fourths of the local population opposed to the project (Smith-Estrada 2011). This “Estonian Phosphate War” or “Phosphate Spring” (Sikk and Andersen 2009) was followed by Estonia’s Green Movement and later linked to assessments of ecological efficiencies associated with shale oil co-mining; shale oil may be produced together with carbon-containing phosphate in the same region.

Nevertheless, 23 countries currently produce marketable phosphate rock on a larger scale (Jasinski 2022), although the data must be interpreted against the backdrop of mineral phosphate rock as a low-cost commodity. Given a high price of US$200/ton for marketable mineral phosphorus, each world citizen consumes phosphorus at a cost of about US$6 per year. Furthermore, given that energy costs per world citizen are of the magnitude of US$1000, a doubling or even a tenfold increase in the price of phosphate rock would, ceteris paribus, i.e., if phosphorus were to become scarce, not cause a collapse of the global food supply and the global economy. Although we do not know the type of mathematical functions, there is evidence that reserves and resources increase (positively) non-linearly with declining cutoff grades (Lasky 1950).

### The scarcity fallacy

#### Rationale

The supply–demand relationship is the core of classical economics. Globally, phosphorus is a demand-driven market commodity rather than a supply-driven market entity (Scholz and Wellmer 2013a). Phosphorus production and trade are shaped—like all sectors of the global agrochain (Mooney 2018)—by an oligopolistic structure.

The proponents of mineral scarcity focus almost exclusively on the demand side in relation to population growth, increasing GDP, and thus, increasing consumption and dietary changes, etc. This holds true particularly for the promoters of the Club of Rome (Meadows et al. 1972), who were the masters of system dynamics but, surprisingly, considered the supply side to be static. D. L. Meadows acknowledged the increases of some metal supply by exploration (e.g., by a factor of five), yet the reserves and resources were not viewed as geo-socioeconomic entities, which are subjects of market dynamics, by nature. Thus, they did not acknowledge that price increases and technological developments allow for the economical mining of lower grades and provide an increase in reserves. For some reason, understanding this basic mineral economic knowledge seems to be overly difficult—not only for laypersons and politicians but also even for natural scientists and those in the sustainability sciences (see below). The reserves are considered fixed stocks, such as a pie that becomes smaller and eventually disappears as slices (which are identified today) are taken. This misconception may be linked to a misunderstanding of the statement that Earth’s phosphorus is finite and that phosphate rock is non-renewable.

We assume that there are evolutionary cognitive barriers related to the mineral scarcity fallacy (Klix 1980; Tooby 2018). We may assume that many people tend to confuse the notion of the term “finite” with “limited,” which in everyday language semantically overlaps the term “scarce.” The concept of infinity (as the opposite of limited) is a theoretical one; astronomical data suggest that the universe is finite (Ellis 2003), and thus, the number of phosphorus atoms on Earth is limited. Certainly, it is smaller than $2.2\times {10}^{57}$, which is the maximum “number of atoms in any kind of star” (Hogan 2000). The amount of phosphorus in Earth’s crust is sufficient to provide the annual current rate of consumption for 63 million years (Scholz and Wellmer 2021). What this means is unclear (as Earth’s terrestrial crust has an average thickness of about 35 km). A problem with these data is that average people, who do not work with these issues everyday like astrophysicists do, have insurmountable difficulty understanding intuitively massive numbers of evolutionary and geologic magnitude. Thus, the magnitude of difference between $2.2\times 10\times 57$ and $2.2\times {10}^{57}$ is beyond the average layperson’s—as well as the average scientist’s—comprehension as it is known for long (see Ciccione et al. 2022; Jevons 1871; Wagenaar and Sagaria 1975).

A perplexing approach to modeling scarcity has been provided by Cordell et al. (2009). Locked in by fixed-pie thinking, they took the USGS Commodity Summary 2009 reserve estimate of 15 metric gigatons (or pentagrams; $15\times {10}^{5}$ g) of marketable phosphate rock (Jasinski 2009, which includes 30% P_2_O_5_; marketable phosphate rock includes 13% or phosphorus) as an estimate of the ultimate recoverable resources (URR) of phosphorus. A commodity is an economic good or commercial product of mining companies, and the planning horizon of economic enterprises does not usually extend beyond a century. The URR denotes the amount of phosphorus that humankind has mined and may mine in the future in relation to tens of thousands or hundreds of thousands of years of the human species life cycle.

Starting from the reserves as an estimate of the URR, there are two oversimplified approaches. One is to calculate the “lifetime of reserves,” i.e., to divide the reserves by the annual consumption (R/C). Based on the 2009 data, the lifetime index would be 89.8 years. Thus, we would soon face a Malthusian collapse due to famine. Cordell et al. (2009) took another approach using a reserve data–based Hubbert curve. The Hubbert curve is a model that makes sense for predicting the production curve for certain deposits whose physical boundaries are well-known, such as the guano phosphorus deposits on the 21-${\mathrm{km}}^{2}$ island of Nauru (Déry and Anderson 2007). The knowledge and technology of mining in a location increases with consumption, and, after maximum production, mining becomes more difficult until it becomes uneconomic. Figure 2 presents the production curve, which does not match a logistic curve but rather a concave-increase and a convex-decline curve with several slightly declining plateaus.

Cordell et al. (2009) applied the Hubbert curve to the global consumption of phosphate rock. They used the 2009 reserve data (as a fixed stock) and assumed that this amount should equal the (area of the) integral below the Hubbert curve. And they concluded that this would be the ultimate amount human species may mine in its lifecycle. This “results in a production at peak of 29 MT P/a and a peak year of 2033” (2009, p. 298) and, subsequently, that “phosphate rock … may be depleted in 50–100 years” (2009, p. 292). Another option for applying the Hubbert curve is the direct fitting of the past production curve with a Hubbert curve (without using the reserve data); this would provide an even shorter forecast for a peak than that of Cordell et al. (Déry and Anderson 2007).

Similarly, perplexing is Cordell et al.’s assumption that, if the peak were to be reached globally (which we assume in order to meet the needs of the global population), no efforts would be made to maintain this maximum production level. This would result in a plateau of production values around the peak for a couple of years (Scholz and Wellmer 2013a). Rustad (2012, 2016) investigated whether Hubbert curve–like consumptions could be observed for any of 36 industrially used mineral resources. As the title of his paper “Peak Nothing” unambiguously conveys, none of these minerals’ consumption follows Hubbert linearization (Rustad, 2012, 2016).

#### Reception of the phosphorus scarcity fallacy in science and in practice

Information about phosphorus and the availability and accessibility of minerals, in general, is in need of a clearinghouse of knowledge, and the scarcity fallacy may be seen as one reason. The New York Climate Change Science Clearinghouse is an example of such an institution (Rikert, Khan, Branchini, & Zabel, 2015).

But how has the phosphorus scarcity claim actually been acknowledged? Surprisingly, the erroneous fixed-stock Hubbert modeling is promoted by a wide community of natural and sustainability scientists. This has been shown by a content analysis of Web of Science indexed papers’ statements on scarcity (Scholz and Wellmer 2021). These fallacious statements are also, of course, conveyed to the public by high-ranking newspapers such as the *New York Times* (NYT 2011) and the *Guardian* (Carrington 2019), and *Der Spiegel* news magazine (Schmundt 2010), which are major sources of politicians’ opinion formation.

We should note that practitioners in the field of phosphorus management do not share the scarcity assumption referred to in Table 1. An inquiry of 16 practitioners from the Global TraPs project board (i.e. Global Transdisciplinary Processes on Phosphorus Management; see Scholz et al. 2014a, b; Steiner et al. 2015) found that 15 of 16 responding practitioners consider statement 3 of Table 1 to be absolutely wrong (Scholz and Wellmer 2021). The sole practitioner who shared the scarcity view specialized in phosphorus plant uptake in agriculture. Yet also two others, without mining background, considered the scarcity assumption a scientifically wrong but conveyed that the non-scarcity proposition is in conflict with their “limits of growths” thinking. In general, both among scientists and among practitioners, we may still find a Wordview/Zeitgeist component as it has been described at the beginnings of the environmental movement. Here the distinctions between cornucopians/weak sustainabilists who believe in the “creative powers of technology and free markets to find substitutes for any and all scarce resources” in contrast to neo-Malthusians/strong sustainabilists “stressing the “limited global stockpile” of critical natural resources, from cobalt to petroleum” (Ayres 1993, p. 189) seems to be an important issue.

**Table 1:** Frequency of Web of Science papers on global phosphorus (1) discussing potential scarcity (absolute number of papers on global phosphorus) (2) explicitly mentioning “scarcity/scarce” in connection with resources and/or reserves or (3) explicitly providing an alarmist scarcity statement, and (4) papers on scarcity (Table taken from Scholz and Wellmer 2021.)

	Topic	2000/1999	2005/2004	2010	2015	2020
1	Papers on global phosphorus reserves/resources	2 (13)	5 (31)	16 (37)	24 (30)	14 (50)
2	Potential scarcity explicitly discussed (term “scarcity” appears)	0 (0%)	0(20%)	4(25%)	9(41%)	8 (57%)
3	Refer explicitly to “rapid depletion” or “50–150 years” depletion alarmist statement (share of category 2)	0 (0%)	2 (40%)	9 (56%)	11 (46%)	10 (71%)
4	Number of papers that fulfill criterion of line 2 *and/or* line 3 above	0 (0%)	2 (40%)	9 (56%)	16 (67%)	14 (100%)

#### Possible reasons for the fallacy

To identify further potential reasons for the beliefs in mid- and long-term scarcity, we distinguish between drivers and rationales of laypersons, experts, scientists, and others.

Two cognitive difficulties come into play. The first is that a deposit is considered a material physical entity with fixed boundaries and, thus, as a genuine static entity. This leads to a static metaphor resulting in fixed-stock numbers, which strongly contradicts the idea that the reserves provided by a deposit (and of global phosphorus) are a demand–supply, dynamics-based socio-geological variable that varies over time. As previously noted, if demand increases, so do prices. If prices decline, the cutoff grades increase, and if the prices increase, the cutoff grades decline. Price increases may even allow increased investments for an improved floatation or beneficiation plant (Kawatra and Carlson 2013) and increased total resource-use efficiency (defined as the share that becomes marketable) given the fraction of the initially moved ores containing that mineral (Scholz and Wellmer 2015). This is the direction future sustainable mining should take (De Villiers 2017), although not in the near term, given a low-cost commodity whose high-grade mines have not yet been depleted (as for gold) and that is easily mineable, such as phosphorus. Thus, a mine’s reserves and their values are continuously changing. This can be seen as an economic challenge, particularly for private mining companies, although the paper by Geissler et al. (2015) shows that privately held phosphorus companies operate slightly (but not statistically significantly) better than public companies. Future research might focus on extraction efficiency to a greater extent.

The second of the abovementioned cognitive difficulties is coping with numbers of geological magnitude. The extrapolation from the Nauru guano deposit, the size of which can be visualized as a concrete object (Gigerenzer 1998; Piaget and Inhelder 1956; Scholz 1989), to the world’s resources and sub-resources of Earth’s terrestrial crust, whose magnitude is far beyond human perceptional experience (Resnick et al. 2017), is an example. This is in line with the finding that large time scales (Moser et al. 2012) or small probabilities (Gigerenzer 2015) are rather confusing as most people cannot comprehend such matters without explicit help in their perception.

In addition, a reflection on whether or not and, if so, how much phosphorus might be economically and environmentally mined responsibly from the sea (which covers two thirds of Earth’s crust) and the geological processes that take place there (Pufahl and Groat 2017) have, presumably, not been considered by the vast majority of people.

Let us look at the drivers, i.e., the motivational side, of the scarcity fallacy. One may assume that, with the transition from the hunter-and-gatherer age to the Holocene’s horticultural and agricultural societies, settled tribes in the North had to develop a deep concern about scarcity and the stockpiling of food and fuel (less than 10,000 years ago). Surprisingly, psychological research on the scarcity frame has been rare and has become a “new research area” in evolutionary psychology (Isler 2019). Moreover, current research considers the scarcity (of features) as an opportunity to develop evolutionary advantages or to analyze why the poor, facing scarcity, have a tendency to become obese (Hinsz et al. 2013; Peschel 2021). We suggest that scarcity is not perceived as an evolutionary, unconscious, innate (amygdala-firing) risk, such as potentially venomous spiders or snakes (Kawai 2019; Öhman et al. 2007). Yet stories about panic buying and stockpiling abound not only in times of war and conflict but also at other times; the COVID-19 pandemic initially featured the hoarding of toilet paper and other products, a phenomenon not previously observed in peace time (Sim et al. 2020). Such exceptional scarcity fears are unknown with respect to global mineral-resource scarcity, but we can learn from qualitative analyses (Leung et al. 2021) that anxiety (which has U-shaped relation with analytical thinking in everyday settings; a bit anxiety or concern elicits the analytic mode) induces disordered thinking by strong (social) media effects that increase anxiety to a panic level (Atkinson 1964; Kuhl 2009, 2018; Scholz 1987). Naturally high levels of anxiety hinder reflective thought (Sweeny and Dooley 2017). We assume that the phosphorus scarcity becomes critical when it is linked to apocalyptic arguments featuring phrases such as “a ticking time bomb” or “our supply of mined phosphorus is running out” provided by the abovementioned *NYT* and *Spiegel* articles. Nevertheless, the scarcity argument has the potential to generate fear and unbalance a meaningful dialogue and, thus, may be used to nudge changes in environmental and political thinking and behavior. The following are examples of this.

- Environmental activists focused on the (often necessary) reduction of phosphorus pollution in aquatic systems can use false scarcity arguments with an end-justifies-the-means perspective. Given the Western value system, a false anthropocentric argument that “we are running out of fertilizer and thus losing about half of the global food production” (e.g., when referring to Erisman et al. 2008) may have stronger political efficacy than ecological arguments such as “we have to protect aquatic systems”—at least in Western cultures (White 1967). Note also that the concept of ecosystem functions is genuinely anthropocentric (de Groot, Wilson, & Boumans, 2002).
- Scientists who work on phosphorus recycling for sewage plants often use the short-term scarcity argument of topic 3 in Table 1 as salient in their research proposals or papers to promote the significance and urgency of their own research. This is unfair, and because it is scientists’ duty to acknowledge disciplinary state-of-the-art knowledge, it is dishonest (and could even, perhaps, be interpreted as criminal in certain cases); more succinctly stated, it is a case of scientists attempting to gain advantages for their own research. Hereby, they are, possibly, intentionally misleading public decision-makers by ignoring evidence (e.g., that the current reserves and half of the known resources will provide the needed annual supply for approximately a millennium).
- Politicians who lack precautionary thinking would not care sufficiently about long-term mineral (phosphorus) supply security. Yet responsibility must be taken for developing a closed loop economy for phosphorus because of its essentiality for food production (in addition to its environmental impacts).

We argue that, from both an environmental impact and a long-term resilience perspective, there are more than enough arguments for putting phosphorus at the top of the mineral commodity hierarchy (Wellmer and Scholz 2017). Thus, it is imperative that this is not endangered by “scientifically” incorrect statements that endanger the integrity and credibility of science.

### Coping with Simpson’s paradox of phosphate rock production

We want to end this part with a pseudofallacy. The paradox is that the average ore grades of mined phosphate on Earth increased by 3.2% over three decades (from 1983 to 2013). This seemingly refutes the rule of thumb that the highest ore grades are mined first. This paradoxical (and misleading) statement occurs as the association between “average ore” grade “annual production in all considered countries” is affected by the changing proportion of phosphate rock produced in the countries whose data are used (Alin 2010). The statement—although not wrong—can be used to irritate and mislead people who are concerned about the dwindling amounts of high ore grades. Actually, the paradox appears in data utilized by Steiner et al. (2015, p. 235):

On a global scale, the ore capacity tonnage and average grade of ore mined have increased over the past 30 years from 513 Mt PR-Ore at 14.3% P2O5 in 1983 to 661 Mt PR-Ore at 17.5% P2O5 in 2013.

The analysis was performed by including all (more than a hundred) large operating phosphate mines in 2013 based on industry data, yet this result exemplifies Simpson’s paradox. A standard illustration of Simpson’ paradox is the following. Imagine a company has 100 branches. Now, in a given period, the company’s records show that all branches made a smaller profit in the last accounting period; however, the average profit of all companies increased. How can this be possible? The rationale behind this paradox is that there is at least one company with a high profit rate compared to others whose profit rate declined slightly, yet the relative share of the turnover of this branch increased considerably. Thus, the average profit rate of the whole company (which is the weighted profit rate among all companies) increased.

Actually, the standard illustration (see Steiner et al. 2015; Table 1) is close to the situation in phosphate rock production; Simpson’s paradox emerged from the data of Chinese phosphate rock production. Production in China was low in 1983, with 6.9 Mt PR-Ore (which made 1.3% of the world production) with an ore grade of 23.6 Mt P_2_O_2._ It increased tremendously to 160.3 Mt PR-Ore (which makes 24% of the world production) with a somewhat lower degree ore grade of 21.6% in 2013. As the 160.3 Mt PR PR-Ore produced in China have a degree of 23.6 Mt P_2_O_2_ that is above the average global ore grade of 17.5%, the Simpson’s paradox appears.

## What do transdisciplinary processes need from mineral economics?

### Honest scientific brokership and science as a clearinghouse

Mineral economic management generically and the discussion about phosphorus are subjects of societal discourse on sustainable management on different scales. The Malthusian scarcity assumption for phosphate rock has impacts on key issues of phosphorus management including (i) increasing the total phosphorus use efficiency; (ii) recycling phosphate rock, e.g., by struvite from sewage plants; (iii) increasing agro-use efficiency and avoiding environmental pollution, especially in aquatic systems; and (iv) reducing the environmental impacts of mining. Yet, in addition, there are general concerns including (iv) increasing social justice regarding access to phosphorus use for poor countries and (v) the external costs of mining properly (more than 95% of phosphorus production takes place in developing countries).

Furthermore, there are a number of other fallacies that might become subjects of mutual learning in transdisciplinary discourses. A worthwhile example of how incorrect information can be used as well as misused in the scientific community is the statement on the distribution of phosphate deposits. Misstatements and questionable conclusions read like the following:

The geopolitics surrounding P dependency on only few countries is not being adequately addressed. (Rosemarin and Ekane 2016, p. 266)

Or, similarly, a paper recently published by the high-ranking journal *Nature Communications* included the following:

The newly discovered P reserves being restricted to a small region of the Western Sahara and Morocco. (Alewell et al. 2020)

These examples appear to be misuses of scientific journals related to matters of international political significance. Two concerns regarding the above comment should be noted. First, the Western Sahara is of some political (postcolonial) interest but does not play a significant role in phosphate rock supply security. Between 1984 and 2004, the Sahara resource increased from 0.95 billion cubic meters PR-Ore to 1.11 billion. The Moroccan (ex-Sahara) tonnage increased from 54.97 billion to 84.39 billion. Thus, the share of Western Saharan resources fell from 1.7% in 1984 to 1.3% in 2004 (OCP 2005). According to the producing company (OCP), the resources in the Western Saharan deposits did not increase above 2% in the 2011 upgrading (Bloomberg Businessweek 2010; Jasinski 2012; Lowe, 2010; Scholz & Wellmer, 2016; van Kauwenbergh 2010). Furthermore, new discoveries and assessments that transfer occurrences to deposits and reserves take place globally. According to industry information, phosphate rock was produced in 37 countries in 2019 (CRU), so at least those must have reserves. In the *Handbook of Exploration and Environmental Geochemistry*, we find a chapter by Chernoff and Orris (2002) that lists 1403 igneous and sedimentary deposits located in 102 countries and 231 guano or guano-related deposits in these countries and, additionally, in 12 small states. Thus, practically speaking, the majority of countries have PR resources. If we look at the production side, phosphorus holds the 28th position (counting from the lowest to the highest HHI; i.e., Herfindahl–Hirschman Index) of 53 commodities, which is close to the median value (Scholz and Wellmer 2021).

All these questions involve in-depth knowledge in regard to mineral economics on both on a scientific and local (community) level. As outlined by Scholz and Wellmer (Scholz and Wellmer 2021), the life cycle community incorporates scarcity assumption in its models of environmental evaluation. These LCA scores are used often in a transdisciplinary setting. For instance, LCA scores can be considered to decide what policy options are selected for supporting research (on recycling) or what taxes could be applied to fertilizers if its (over-)application is environmentally harmful (Scholz and Geissler 2018). Likewise, as environmental economics is part of the interdisciplinary folder, the assessment of external costs (and their relations to various technologies applied) of a specific local mine calls for sound mineral economics knowledge. Thus, knowledge from mineral economics is needed in many transdisciplinary processes—but it must be considered and applied in an honest and unbiased manner.

### Coping with fallacies calls for cognitive didactics and “Eurekas!”

Transdisciplinary processes related to mineral economics require that the appropriate knowledge be used. Moreover, they call for that knowledge to be used correctly, appropriately, understandably, and unambiguously, which leads to cognitive didactics (Nothbaum and Scholz 1995). Thus far, this concept has not been widely applied, yet it may become crucial if the interactions of more than two variables are essential. The art of cognitive didactics is that a decision-maker acquires information for essential decisions in the form of a representation that allows him or her to understand the key relations and data involved in a problem (Scholz 1999). For instance, in Simpson’s paradox (Alin 2010; Wagner 1982), the relations between two variables (i.e., the decrease of average ore grades in all mines worldwide with the variables $x$ as “ore grade” and $y$ as “time”) are reversed after considering a third variable (i.e., $z$ as “change of relative share of a production of countries”) (Alin 2010). China’s immense increase in the production of higher-than-average ore grade phosphate rock increases global ore grades as slight declines in other countries are not sufficiently significant.

**Fig. 3:**
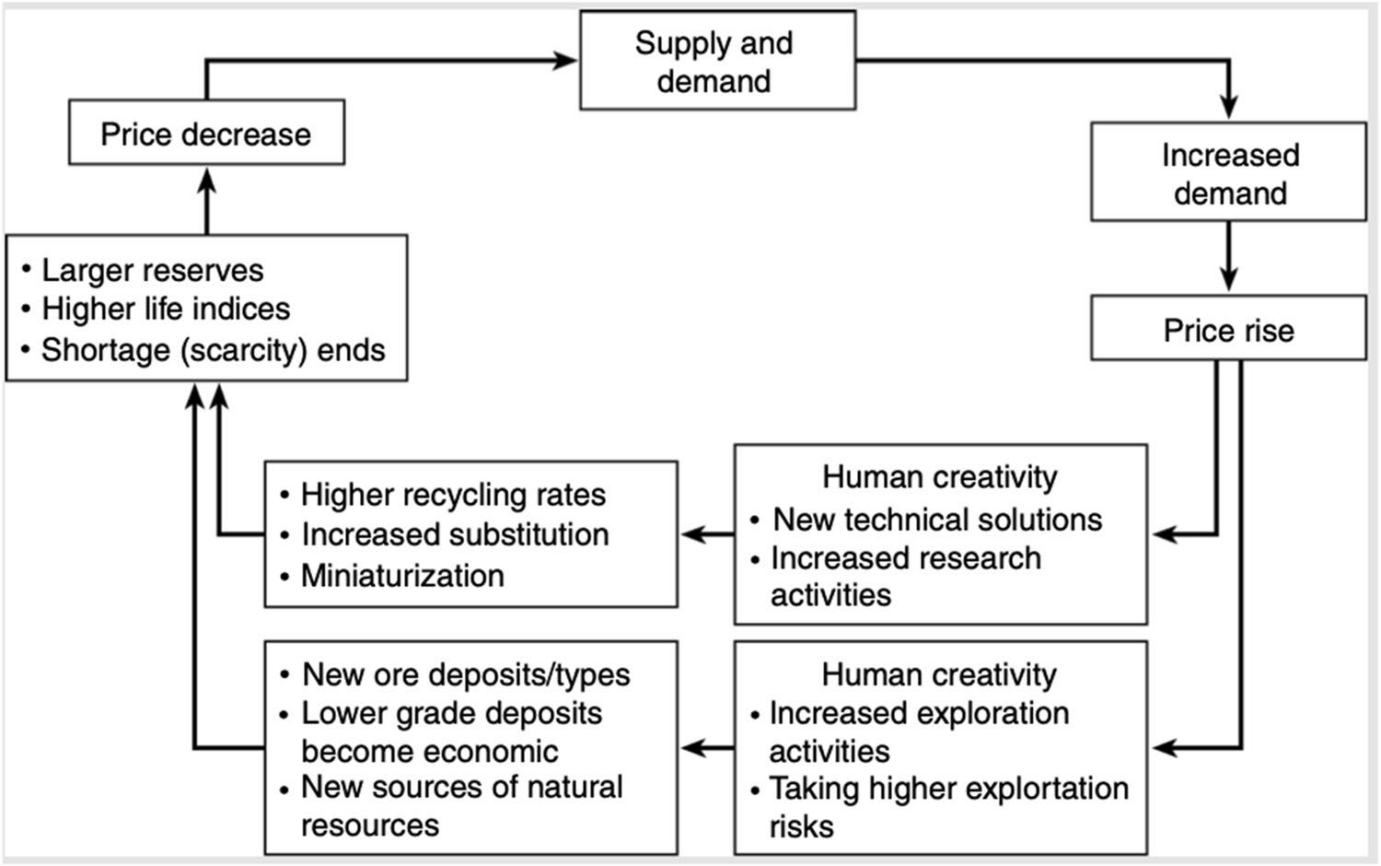
The feedback control cycle of mineral resource management (Wellmer and Becker-Platen 2002); figure taken from Scholz (2011)

Another example of missing cognitive support is the “if prices rise, so do stocks” rule. Here, the cognitive barrier is that prices rise (variable $y$) over time (variable $x$) and affect a set of other variables. The decline in the ore grade with economic production, given an over-proportional increase of additional reserves (variable ${z}^{1}$), the increase of exploration efforts ${z}^{2}$, or recycling ${z}^{3}$ because the mining of a mineral promises a higher return on investment, may be taken as examples. Relatedly, Wellmer and Becker-Platen (2002) developed the feedback control cycle of mineral resource management (see Fig. 3). The graphic explains the “Price rise” → “Larger reserves” relationship by embedding it in the rationale of “supply and demand” logic. The feedback control cycle is easy to understand, works without formulas, explains the basic rules of the game, and thereby, has the potential to elicit a Eureka! effect.

Science is a public good, at least to the extent that most universities and large amounts of research are funded by the taxpayer (for a deeper discussion of this partly disputed issue, see (Scholz 2020)). If we follow this concept, science receives a kind of clearinghouse function. Scientists who represent public institutions should act neither as normatively driven activists nor as profit-oriented, contract-based knowledge workers but rather as honest knowledge brokers.

The phosphorus scarcity question or the mineral scarcity question can be a good example. However, in addition, the Simpson’s paradox–based increase of ore grades could be misused in political contexts; consider the repeated incorrect statement that economically mineable phosphate rock can be found in only a few countries. For developing meaningful or, to avoid the term rational (Pinker 2021), better decisions as both for high level science and honest knowledge brokership.

There is another set of fallacies, which can be found in many publications, from the field of mineral economics that are in need of correction on a large scale. That phosphate occurrences and potential mines are located in only a few countries is one example. Moreover, many papers that consider the phosphate price peak to be a trigger of the 2007/2008 world economic food crisis can be found. There are those who think that high heavy metal or radionuclide concentrations imply that phosphate rock might not become reserves at some point. The option of co-mining with heavy metals or trace elements such as beryllium–scandium or rare earth minerals (Chen and Graedel 2015) or uranium (Shang et al. 2021; Steiner et al. 2020) has been used on a large scale since the mid-1990s in the USA (Haneklaus et al. 2017). In addition, amelioration of phosphate rock quality has been achieved by extracting considerable amounts using new floatation technologies (Benredjem et al. 2016). The relatively inexpensive extraction of cadmium in wet phosphoric acid production (Cichy et al. 2014; Kouzbour et al. 2019) may also be considered an option and should be discussed in transdisciplinary processes since commercial and public interests are intertwined in these topics. Understandably, all these challenges need mineral science and mineral economics knowledge; therefore, new forms of interface management between science and practice are necessary.

### The example of the Global TraPs project

We end by presenting a successful example of a transdisciplinary project in mineral resources management. The Global TraPs project, a process that included about the same number of scientists and practitioners at all levels, has been mentioned above. More than 300 practitioners and scientists participated intensively in the workshops and interactions for this 2011–2014 process. Global TraPs was supported by research funds, UNESCO, mining companies, fertilizer associations, and the International Fertilizer Development Center (IFDC, USA), of which the practice leader, Amit Roy, was the president, as well as by ETH Zürich (Switzerland) and the Fraunhofer-Gesellschaft (Germany), the institutions of the science leader Roland Scholz. The participation of Greenpeace and of Indian Smallholder Farmers or Kenya National Farmers’ Federation shows that a balance among stakeholders was reached (see the organizational charts in: Scholz et al. 2015; Scholz et al. 2014a, b; Scholz, Ulrich, Eilittä, & Roy, 2013).

There has been a thorough, trustful, mutual-learning process that concluded with a comprehensive system analysis of the nodes of the supply chain (i.e., exploration, mining, processing, use, recycling, and trade and finance as overarching issues). This analysis was documented in the book *Sustainable Phosphorus Management: A Global Transdisciplinary Roadmap.* The chapters were jointly written by 18 practitioners and 23 scientists and based on numerous reviews and feedback from the fields of science and practice. Global TraPs provided a roadmap for reducing environmental pollution, for increasing efficiency and avoiding losses, for identifying secondary mining opportunities, and for recycling and resource conservation options, among others.

Follow-up projects, such as the “Kenya smallholder access to phosphorus” (Chebet et al. 2019; Njoroge et al. 2015), dealt with the constraints of accessing phosphorus in farmers’ crop cycles. In two sequential transdisciplinary studies at the interface, scientists and practice representatives from all key stakeholder groups of the supply chain (including local banks) participated. Moreover, 201 farmers participated in two additional sequential experimental studies about applying phosphate fertilizers according to soil testing–based recommendations and participated in a transdisciplinary process including scientists from local universities. In both studies, yields increased by about 40% (from 4.5 to 7.0 tons dry weight maize per hectare). Mineral economics knowledge was challenged by analyzing and evaluating the double input system by state and commercial companies.

## Conclusions

As aptly delineated by John Tilton’s introductory quotation, mineral economics is a traversal discipline. It relates to different topics from exploration, mining, processing, logistics, efficacy and efficiency of use, and environmental impacts and their costs. It is an overarching topic on the availability of minerals and their economic access as it focuses on the material side of the production, distribution, and consumption of mineral commodities and, most recently, on the topic of human and environmental health. Mineral economics particularly describes mechanisms such as market dynamics, rivalry, excludability, taxation, and other means of including external costs.

In the presented conception of science–practice collaboration-based transdisciplinarity (see Fig. 1), targeted interdisciplinarity is a prerequisite for undertaking transdisciplinarity. Taking a critical outsider view, one may state that mineral economists, with some exceptions, have not succeeded in a resounding way to develop or participate in the various processes toward sustainable mineral resource management (Brundtland et al. 1987). First, we conclude that one reason may be seen in a camp building between “green sustainability thinkers” and mineral economists who follow a traditional multidisciplinary path. From a strong sustainability scientist and environmental scientist perspective, (mineral) economists are often viewed as profit-maximizing modelers who ignore principles such as “keeping the natural capital intact (over time)” (Holland 1997, p. 119) or the need to preserve natural capital (Dedeurwaerdere 2014; Goodland and Daly 1996) and who believe in the substitutability of any natural capital. The case of phosphorus shows that the latter is not possible; this was acknowledged by the paper “Putting phosphorus first: The need to know and right to know call for a revised hierarchy of natural resources” (Wellmer and Scholz 2017). One step toward bridging the gap between the camps was taken though, presumably, many more steps have to be done.

Second, the physical scarcity claim seems to be deeply established in the mindsets of many sustainability researchers. This is also outlined by a survey presented in Fig. 2. The basic rule, “if prices increase, so do stocks,” and Wellmer and colleagues’ feedback control cycle of mineral resource management (see Fig. 3; Scholz 2011; Wellmer and Becker-Platen 2002; Wellmer and Dahlheimer 2012; Wellmer and Hakelüken 2015) have not yet been sufficiently acknowledged, understood, cited, and conceptually incorporated The Club of Rome’s “limits of growth” metaphor and the conception of mineral reserves as a fixed pie or “Eiserne Reserve” (i.e., iron reserve; a German phrasing of emergengy rations which emerged in Military affairs for food and munition; Hildebrandt 1820) suggest a fixed stockpile. It is (implicitly) assumed here that this proportion is constantly decreasing. We further showed that a fixed-pie assumption was used in the community of life cycle modelers to operationalize physical scarcity threats as a concept complementary to environmental impact. The economic idea that a mineral commodity such as marketable phosphate rock can be produced as long as we can pay for it was abandoned. From an environmental and sustainability perspective, the challenge here would be to properly include the external costs. Based on the seminal concept of cumulative availability curves (Tilton et al. 2018; Yaksic and Tilton 2009), a further step can be taken toward an interdisciplinary mineral resource management incorporating sustainability and mineral economic considerations.

Third, sustainability science takes an intergenerational perspective. This exceeds 100 years, which may be seen as the maximum planning horizon of economic considerations in mining. Actually, the time window for having data on market economics, a source of validating economic models, is about 150–200 years. If we consider the dynamics of reserves and resources, a time window of more than 1000 years opens. In our opinion, it is a fundamental theory of knowledge and a basic ethical (thus, also a cultural and religious) question whether we should think or even take on a moral (asymmetric) responsibility related to such a long-term perspective (Persson and Savulescu 2019; Wilson 1999). Nuclear waste management, for example, poses similar questions that may be viewed differently by the two camps.

Fourth, as discussed, mineral economics is facing the situation that highly specialized scientists who work on specific topics present provocative statements on mineral scarcity that attract attention. The statement, We are facing an “immediate threat of … a P limitation” (Alewell et al. 2020), published in the top-ranking journal *Nature Communications*, may be taken as an example. This is endangering the presented vision that science should become a clearinghouse of science.

Conclusion 1: Mineral economics is a genuinely multidisciplinary field that is challenged to build bridges to sound, comprehensive, discipline-based, interdisciplinary, and transdisciplinary mineral-resource modeling and management.

The second conclusion refers to the mineral economics–society relationship. Unfortunately, social distrust in industries, and particularly the mining industry’s sensitivity and willingness to make a commitment to corporate social responsibility, is low and dominated by the economic survival instinct (Owen and Kemp 2013). Thus, the development of trust and belief in companies representing the mining industry, which is still a “conservative industry following a slow pace of changing” (Hitch and Barakos 2021), is not only part of a political agenda but also part of the economic framing of political, socio-normative, and societal regulatory frames and regimes of mining. This poses the challenge that mineral economics also has to descend from the ivory tower and include the interface not only between other scientific disciplines and the mining industry but also with the broad range of societal stakeholders.

We have stressed honest knowledge brokership, the role of science as a clearinghouse in a world shaped increasingly by fake news. We believe that mineral economics knowledge is needed at all scales. The local, community level relates to the mining process, e.g., when properly including social and environmental costs in such a way that fair and equitable solutions can emerge. This was outlined more than 20 years ago in a convincing way (MMSD 2002). The national level may be linked to industries such as waste management in iron and the steel industry (Matsubae-Yokoyama et al. 2009; Nättorp et al. 2019) or to the design of alloys that may enable different use efficiencies (Raabe et al., 2019). The global level has been addressed in this paper and relates to short- and long-term access, environmental and other costs, social justice regarding access (which may become a subject of welfare functions), the potential as well as limits of technology development, and our knowledge about what parts of our geopotential may become reserves and why, how, and when. Global TraPs as a global project was based on a transdisciplinary process; here, economic knowledge played an important role. Thus, one may state that mineral scientists with a strong economic mindset, such as Fred Wellmer, pioneered transdisciplinary ventures by helping to avoid misunderstandings in both interdisciplinary settings and in practice. There has also been much learning from practice on the side of practice. Thus, practice needs mineral economics knowledge so that mineral economists can learn from practice in different ways.

Conclusion 2: Mineral economics and mineral economists pioneered transdisciplinary ventures by helping to avoid misunderstandings and finding substantiated pathways to social licensing. This calls for the basic principles of resources dynamics and economics to be conveyed in an understandable way in order to develop sufficient environmental literacy. Practice knowledge on market mechanisms, consumers and mining companies’ behaviors, political regimes, etc. may promote groundbreaking ideas with the potential to lead to sustainable mineral economics.

## Data Availability

The source of all data, figures etc.
